# Lectures on the Principles of Surgery

**Published:** 1885-03

**Authors:** 


**Lectures on the Principles of Surgery.** By W. H. VAN BUREN, M. D., LL. D., formerly Professor of Principles and Practice of Surgery in the Bellevue Hospital Medical College. Edited by LEWIS A. STIMPSON, M. D., Professor of Physiology and Clinical Surgery, University City of New York. New York: D. Appleton & Co., 1, 3 and 5 Bond street. 1884.

This volume is one which will be gladly welcomed, not only by those who have enjoyed Dr. Van Buren's teaching, but also by the profession, by whom the practical value of the work will be appreciated. It is published from the manuscript which the distinguished professor employed in his lectures, with but few changes and without additions; while it is not, therefore, a complete treatise on the principles of surgery, it is that presentation of them which he, in his large experience, thought best fitted to be of service and of practical value.

